# New mutations in flagellar motors identified by whole genome sequencing in *Chlamydomonas*

**DOI:** 10.1186/2046-2530-2-14

**Published:** 2013-10-30

**Authors:** Huawen Lin, Nicholas P Nauman, Alison J Albee, Silas Hsu, Susan K Dutcher

**Affiliations:** 1 Department of Genetics, Washington University, 660 South Euclid Avenue, St Louis, MO 63110, USA

**Keywords:** Kinesin-2, Cytoplasmic dynein, IFT81, Ciliary assembly, IFT recycling, Whole genome sequencing

## Abstract

**Background:**

The building of a cilium or flagellum requires molecular motors and associated proteins that allow the relocation of proteins from the cell body to the distal end and the return of proteins to the cell body in a process termed intraflagellar transport (IFT). IFT trains are carried out by kinesin and back to the cell body by dynein.

**Methods:**

We used whole genome sequencing to identify the causative mutations for two temperature-sensitive flagellar assembly mutants in *Chlamydomonas* and validated the changes using reversion analysis. We examined the effect of these mutations on the localization of IFT81, an IFT complex B protein, the cytoplasmic dynein heavy chain (DHC1b), and the dynein light intermediate chain (D1bLIC).

**Results:**

The strains, *fla18* and *fla24*, have mutations in kinesin-2 and cytoplasmic dynein, respectively. The *fla18* mutation alters the same glutamic acid (E_24_G) mutated in the *fla10-14* allele (E_24_K). The *fla18* strain loses flagella at 32?C more rapidly than the E_24_K allele but less rapidly than the *fla10-1* allele. The *fla18* mutant loses its flagella by detachment rather than by shortening. The *fla24* mutation falls in cytoplasmic dynein and changes a completely conserved amino acid (L_3243_P) in an alpha helix in the AAA5 domain. The *fla24* mutant loses its flagella by shortening within 6 hours at 32?C. DHC1b protein is reduced by 18-fold and D1bLIC is reduced by 16-fold at 21?C compared to wild-type cells. We identified two pseudorevertants (L_3243_S and L_3243_R), which remain flagellated at 32?C. Although *fla24* cells assemble full-length flagella at 21?C, IFT81 protein localization is dramatically altered. Instead of localizing at the basal body and along the flagella, IFT81 is concentrated at the proximal end of the flagella. The pseudorevertants show wild-type IFT81 localization at 21?C, but proximal end localization of IFT81 at 32?C.

**Conclusions:**

The change in the AAA5 domain of the cytoplasmic dynein in *fla24* may block the recycling of IFT trains after retrograde transport. It is clear that different alleles in the flagellar motors reveal different functions and roles. Multiple alleles will be important for understanding structure-function relationships.

## Background

The building of a cilium or flagellum requires molecular motors and associated proteins in a process termed intraflagellar transport (IFT). IFT was first described as a bidirectional movement of 'granule-like? particles along the axoneme in *Chlamydomonas reinhardtii*[1]*.* Concurrently, a novel heterotrimeric kinesin was isolated from sea urchin embryos [2]. A temperature-sensitive mutation in the *Chlamydomonas FLA10* gene shows it is needed for flagellar assembly [3], and that IFT is dependent upon FLA10 [4]. *FLA10* encodes a subunit of the heterotrimeric kinesin first found in sea urchins [5,6]. The *Chlamydomonas FLA8* and *FLA3* genes encode the other kinesin-2 motor subunit and the kinesin-associated protein (KAP) subunit, respectively [7,8]. The IFT trains are composed of at least 19 proteins, which fall into two complexes, A and B, which are dissociated by salt [5,6]. Complex B contributes to anterograde transport away from the cell body [5], and complex A is involved in retrograde transport toward the cell body [9-11]. Anterograde movement requires kinesin-2 and retrograde movement requires cytoplasmic dynein. At the tip, the anterograde IFT particles rearrange into new trains with a different shape and size for retrograde IFT [12]. This simple picture is made more complex by examining the behavior of the BBSome in *Caenorhabditis elegans*[13]. The BBSome is a complex of seven proteins [14] that is postulated to be involved in the import of G protein coupled receptors in mammalian cells [15,16] and the export of cycling proteins in *Chlamydomonas*[17]. The BBSome may regulate anterograde IFT assembly and then rearrangement at the tip [13]. Cargo-specific adapter proteins may be important for IFT transport of cargo [18]. IFT is essential for mammalian development as mutants in various IFT proteins are lethal in mice (reviewed in Eggenschwiler and Anderson [19]). Mutations in several IFT proteins and cytoplasmic dynein cause asphyxiating thoracic dystrophy in humans [20-23].

A collection of temperature-sensitive mutants in *Chlamydomonas* that assemble flagella at the permissive temperature of 21?C, but lack flagella at the restrictive temperature of 32?C (Table?1) provides an important resource for the analysis of flagellar assembly [11,24,25]. Since many conditional mutants have reduced but sufficient function at the permissive temperature, this collection offers the opportunity to examine IFT in assembled flagella at the permissive temperature to ask about the effects of reduced function. For example, the temperature-sensitive allele in IFT172 suggests a role in remodeling IFT at the tip [26]. IFT is required to transport many of the flagellar proteins from the cytoplasm to the flagella. These include the inner dynein arm protein p28 that fails to be imported in the *fla10-1* mutant [6]. Recent isobaric tags for relative and absolute quantitation (iTRAQ) experiments suggest that numerous proteins accumulate or are depleted in the presence of a mutant cytoplasmic dynein even when the length of the flagella has not changed [27], which shows the importance of retrograde movement for moving proteins back to the cell body.

**Table 1:** Phenotypes of intraflagellar transport (IFT) mutants and depletion

**Conditional IFT mutants in *Chlamydomonas***				
**Gene**	**IFT phenotype at 21?C**	**Protein**	**Other names**	**Reference(s)**
*FLA1*	Reduced anterograde velocity and train number	Kinesin-2 motor subunit	*fla8-2*	[8,11,24,25]
*FLA2*	Reduced retrograde velocity and train number	Unknown		[24,25,28]
*FLA3*	Reduced frequency of anterograde trains	KAP subunit of kinesin-2		[7,11,24,25]
*FLA4*	Wild-type	Unknown		[24,25]
*FLA5*	Wild-type	Unknown		[11,24,25]
*FLA6*	Not tested	No longer extant		[24,25]
*FLA7*	Not tested	Kinesin-2 motor subunit	*fla10-14*	[8,24,25,29]
*FLA8*	Anterograde	Kinesin-2 motor subunit	*fla8-1*	[8,25,30]
*FLA9*	Wild-type	IFT81		[25], (unpublished data)
*FLA10*	Reduced anterograde velocity and train number	Kinesin-2 motor subunit	*fla10-1*	[3,25,30]
*FLA11*	Reduced retrograde velocity and train number	IFT172	*fla11-1*	[11,25,26]
*FLA12*	Faster anterograde and retrograde velocities	Unknown		[11,29]
*FLA13*	Not tested	Unknown		[11,29]
*FLA15*	Reduced retrograde velocity and train number	IFT139		[11,31]
*FLA16*	Reduced retrograde velocity and train number	IFT144	*fla17-2*	[11,31]
*FLA17*	Reduced retrograde velocity and train number	IFT144		[11,31]
*FLA18*	Reduced anterograde velocity and train number	Kinesin-2 motor	*fla10-16*	[11], this report
*FLA21*	Wild-type	Unknown		[11]
*FLA24*	Reduced retrograde velocity and train number	DHC1b		[11], this report
*FLA27*	Decreased anterograde train number with wild-type velocity	Unknown		[11]
*FLA28*	Reduced anterograde velocity and train number	Unknown		[11]
*DHC1b*	Reduced retrograde velocity and train number	Cytoplasmic dynein	*dhc1b*^*ts*^*; dhcb1-3*	[11,27,32]
**Nonconditional IFT mutants in *Chlamydomonas***				
**Gene**	**Phenotype**	**Protein**		
*FLA8*	Aflagellate	Kinesin-2 motor subunit	*fla8-3*	[30]
*FLA10*	Aflagellate	Kinesin-2 motor subunit	*fla10-2*	[33]
*FLA14*	Short flagella	LC8		[34]
*IFT46*	Short, paralyzed flagella	IFT46		[18,35,36]
*BLD1*	Aflagellate	IFT52		[37,38]
*IFT80*	Aflagellate	IFT80		[30]
*IFT88*	Very short flagella	IFT88		[36,39,40]
*IFT121*	Aflagellate	IFT121		[41]
*IFT122*	Aflagellate	IFT122		
*DHC1b/SPT1*	Very short flagella	DHC1b	*dhcb1; spt1-1; spt1-2*	[9,10]
*D1bLIC*	Variable flagellar length	D1bLIC	*D1blic-1*	[42]
**RNAi depletion IFT strains in *Chlamydomonas***				
*IFT27*	Short flagella	Rab-like		[43]
*IFT70*	Short flagella	IFT70/Dyf1		[44]

IFT, intraflagellar transport; KAP, kinesin-associated protein.

The role of IFT differs between different axonemal proteins/cargos. Piperno *et al*[6] used temporary dikaryons, which are formed by the mating of two parental cells, to examine the kinetics and localization of proteins using antibodies to proteins in axonemal structures. The parental strains both carried the temperature-sensitive *fla10-1* mutation in kinesin-2 [3] that stops IFT within 30 minutes after the shift to the restrictive temperature. One parent is otherwise wild-type, while the other parent has either an *oda6* mutation that blocks assembly of the outer dynein arms [45] or an *ida4* mutation that blocks assembly of a subset of inner dynein arms [46]. In *ida4* x wild-type dikaryons at 21?C, IDA4 appear at the distal end of the mutant flagella by antibody staining and staining moved towards the proximal end with time after mating. In *oda6* x wild-type dikaryons, ODA6 behave very differently. Staining appears along the entire length of the flagella 6 minutes after mating. The intensity increased with time. To test the role of IFT in the incorporation of dynein arm proteins, the parental cells were shifted to 32?C for 30 minutes to inactivate kinesin-2. The incorporation of IDA4 was blocked at the restrictive temperature, while ODA6 continued to be incorporated. Thus, the outer dynein arms appear to enter by diffusion or by a different motor complex [6], while the entry of the inner arm component requires kinesin-2. Transport of outer dynein arms also requires an adapter between the dynein arms and IFT. ODA16 functions as a cargo-specific adaptor between IFT particles and outer row dynein needed for efficient dynein transport into the flagellar compartment, as shown by its localization and interactions by immunoprecipitation and yeast two-hybrid experiments [18]. Recent results suggest that transport of tubulin into cilia is mediated by a weak affinity between tubulin and IFT81 and IFT74 [47].

Analysis of IFT using differential interference contrast (DIC) optics and kymographs showed that six of these conditional mutants have defects in the number of anterograde IFT particles or their velocity at 21?C, six have defects in either retrograde IFT particle number or velocity at 21?C, and four have no change in IFT particle number or velocity at 21?C (Table?1). As genes have been identified, it is clear that alleles in the same gene have slightly different properties; these differences must reflect the properties of the mutant alleles and not the function of the gene. For example, the *fla1* and *fla8* mutants both encode the other motor subunit of kinesin-2 [8] but show differences in the behavior of IFT particles [11]. These differences may reflect the degree of activity/concentration of the mutant proteins at the permissive temperature.

In *Chlamydomonas,* seven genes needed for intraflagellar transport have been identified by conditional alleles (Table?1). Nonconditional mutations in nine additional genes as well as in three of the genes with conditional alleles have been identified. RNA depletion of two IFT genes has been analyzed in *Chlamydomonas* and result in short flagella (Table?1), which may occur because there is only partial knockdown of the genes*.* Eighteen of the IFT and motor protein genes have mutants or depletion results. In this report, we employed whole genome sequencing to identify the *FLA18* and *FLA24* genes*.* These genes encode a new allele in the FLA10 kinesin-2 motor subunit and a new allele in the cytoplasmic dynein DHC1b, respectively.

## Methods

### Strains and culture conditions

Strains were obtained from the *Chlamydomonas* Resource Center (University of Minnesota, St Paul, MN, USA): *fla18*, CC-3864; *fla24*, CC-3866; 137M, CC-124; 137P, CC-125; S1C5, CC-1952; and S1D2, CC-2290. Each *fla* strain was backcrossed three times to either 137P or 137M strains to remove any unlinked modifiers.

### Whole genome sequencing

*Chlamydomonas* genomic DNA preparation for whole genome sequencing was prepared as described previously [30]. Three micrograms of DNA were submitted to Genome Technology Access Core (St Louis, MO, USA) for library construction, Illumina sequencing (San Diego, CA, USA), and initial data analysis. For multiplex Illumina sequencing, 7-nucleotide indexes were added to individual DNAs during the library construction before the samples were subjected to sequencing. The *fla18* and *fla24* samples were tagged with TGAGGTT and GCTTAGA, respectively, and shared the same sequencing lane with two other samples. All resulting sequencing data were de-multiplexed before being subjected to sequence alignment and SNP calling.

### dCAPS markers and segregation analysis

Restriction enzymes that provide differences between mutant and wild-type alleles are listed in Additional file 1: Table S1. For *fla24*, NEBCutter (New England BioLabs, Ipswich, MA, USA) was used to find the appropriate restriction enzyme. However, no restriction enzyme distinguishes between CC-125 and *fla18*. A dCAPS marker was designed using dCAPS Finder 2.0 (Washington University, St Louis, MO, USA) [48]. A forward primer (fla18-dcapF) introduces a mismatch immediately upstream of the point mutation that creates an *Mbo*II recognition site in the wild-type PCR product (GA**A**GA(N)_8_) but not in the *fla18* PCR product (GA**G**GA(N)_8_). The 132 bp PCR product, when digested with *Mbo*II, generates 102 bp and 30 bp fragments from wild-type but is uncut in *fla18*.

### Flagellar isolation

Flagella were isolated as described previously [49,50] with the addition of Protease Arrest (G-Biosciences, St Louis, MO, USA).

### Flagellar counts

Cells were grown overnight in a 21?C lighted incubator to a density of approximately 2 ? 10^6^ cells/ml. Cells were then transferred to a 32?C lighted incubator and samples were taken every hour. Samples were prepared by spotting 19 ?L of cells onto a microscope slide and adding 1 ?L of 2% glutaraldehyde in 0.1 M phosphate buffer (pH 7.0) directly to the spotted cells. A total of 200 cells from each strain at each time point were scored using phase optics (40x) for the presence or absence of flagella. Flagellar length was monitored by immunofluorescence with monoclonal antibody to acetylated ?-tubulin (Sigma-Aldrich, St Louis, MO, USA) at a dilution of 1:1,000.

### Immunoblots and immunofluorescence

The antibodies were: ift81.3 (a gift from Dr Doug Cole) for immunofluorescence at 1:200; ?-tubulin (DM1? from Sigma-Aldrich) used for immunoblots at 1:5,000, DHC1b and D1bLIC (a gift from Dr George Witman, University of Massachusetts, Worcester, MA, USA) were used for immunoblots at 1:2,000 and for immunofluorescence at 1:100; and ?-tubulin was used at 1:500 for immunofluorescence [51]. For immunoblots, flagellar proteins were isolated and resuspended in HEPES/Sr/DTT/sucrose buffer [52]. All the protein samples were stored at -80?C before use. Protein concentrations were ascertained by using Bio-Rad protein assay (Bio-Rad, Hercules, CA, USA) following the manufacturer?s instruction. The proteins were boiled for 5 minutes and centrifuged for 1 minute before loading onto the gel. The proteins were size-fractionated on SDS-PAGE minigels (1.0 mm thick, 6% acrylamide gel (for DHC1b) or 10% acrylamide gel (for D1bLIC) prepared from 30% acrylamide and bis-acrylamide solution, 29:1 (BioRad, Hercules, CA, USA)) and transferred to Immobilon-P PVDF membranes (EMD Millipore, Billerica, MA, USA) in 25 mM Tris, 192 mM glycine buffer containing 20% methanol at 62v for one hour. The incubation with primary antibody was incubated overnight at 4?C. The blots were washed three times for 10 minutes each in PBST. The secondary antibody was incubated at room temperature for 1 hour. Goat anti-mouse HRP (BioRad) and goat anti-rabbit HRP (Sigma-Aldrich) were used at 1:5,000 dilution. SuperSignal West Femto Chemiluminescent Substrate (Thermo Scientific, Waltham, MA, USA) was used according to the manufacturer?s instructions. The blots were imaged on a FluorChem H2 imager (Alpha Innotech, Santa Clara, CA, USA). Signal quantification analysis was performed by ImageJ (National Institutes of Health (NIH), Bethesda, MD, USA).

For immunofluorescence, Alexa 488 goat anti-rabbit (Invitrogen, Grand Island, NY, USA) and Alexa 594 goat anti-mouse were used at a 1:500 dilution with published protocols [51], except newborn goat serum and fish gelatin were not used. All individual immunofluorescence channels were maintained with the same exposure time throughout the time courses.

## Results

To identify and understand the function of genes that can be mutated to a temperature-sensitive flagellar assembly phenotype, we are using whole genome sequencing. Mutants with an anterograde IFT defect *(fla18*) and a retrograde IFT defect (*fla24*) were chosen for analysis [11]. Each mutant was crossed to wild-type (CC-124 or CC-125) to verify that the phenotype segregated as a single mutation in meiotic progeny. In 56 and 130 tetrads, respectively, the aflagellate phenotype at 32?C segregated two wild-type and two mutant progeny, which suggests a single mutation or several tightly linked mutations. Each mutant was subjected to whole genome sequencing. The coverage ranged from 51-fold for *fla24* to 94-fold for *fla18* (Table?2).

**Table 2:** **Changes in *fla18 *** **and *fla24 *** **strains by whole genome sequencing**

**Mutant strain**	**Sequencing reads (101 bp, paired-ended)**	**Aligned reads % (coverage)**	**Number of changes across the genome**	**Chromosome**	**Position**	**Change**
*fla18*	157313244	70.7% (94x)	892	17	4328331	gAg/gGg E to G
*fla24*	63540680	95.3% (51x)	62	6	165063	cTg/cCg L to P

### *FLA18* encodes a kinesin-2 subunit

The *fla18* mutant strain was crossed to the highly polymorphic strain S1C5 and one *fla18* meiotic progeny was subjected to whole genome sequencing [8]. About 71% of the 101 bp reads align to the reference genome (Table?2). A total of 43,103 SNPs/indels unique to the *fla18* mutant strain were found after subtracting the SNPs/indels found in 15 other *Chlamydomonas* strains [53]. Among this set of SNPs/indels, 892 changes map to exons or intron/exon boundaries when synonymous changes are excluded (Table?2).

In our study of transcript levels following pH shock at various time points during flagellar assembly [54], we found that all of the IFT genes are upregulated at least 2.5-fold within 1 hour of flagellar amputation. Given the *fla18* mutant strain has a defect in anterograde IFT [11], we hypothesized that the *FLA18* gene is likely to be among the 1,850 upregulated genes. A comparison between the list of genes that have SNPs/indels in *fla18* and the list of upregulated genes identifies 85 SNPs/indels in 59 genes. One of the changes is a glutamic acid (GAG) to glycine (GGG) in the *FLA10* kinesin (E_24_G, Table?3). In the *fla10-14* mutant strain, the same glutamic acid is replaced by lysine (E_24_K) [8]. We then verified that the temperature-sensitive phenotype was linked to *FLA10* in 20 progeny from a cross of *fla18* x S1C5 (Table?3). The *FLA10* gene is located at 4.43 Mb on chromosome 17. The *fla18* maps 5 map units (mu) from a marker at 4.0 Mb, and markers at 2.23 Mb and 6.8 Mb show weaker linkage (20 and 16 mu, respectively, Table?3). These values conform to other crosses where approximately 100 kb corresponds to 1 mu and indicate that *fla18* is linked to the *FLA10* gene.

**Table 3:** **Primers for mapping of *fla18 *** **in crosses with CC-1952 (S1C5)**

**Primer name**	**Sequence F (5? to 3?)**	**Sequence R (5? to 3?)**	**Product size in *fla18 *** **and restriction enzyme**	**Fla18:S1C5 parental to recombinant progeny**
Ch17-0.5	GCA CAG CTG AAG CGC AAA AGG AAG C	CGT TTC TCG AAC TCA GCC ACT GT	*Hind*III 180 bp	11:9 Unlinked
LC5-2.32	GGA CGG TGG GTA TGC ATT AG	GCT GTC ACT ACG TGG TCT CG	*Msp*I 203 bp	15:5 (20 mu)
Ch17-4.03	ATA TTA CGC CTC TCC GAC AAC AGC	CAG CTT CTT TGT GCG CTT GTA CTT	-271 bp	19:1 (5 mu)
Ch17-6.28	CAT CGA GCT GCT TGG AGG CCA GAT A	CGC TAT ACA CCA CAT AGC GTC GAG	-147 bp	16:4 (16 mu)

Markers without a restriction enzyme generate size polymorphisms due to CA repeats. All reactions were performed with an annealing temperature of 53?C.

To confirm that this mutation is the causative change in *fla18*, we isolated revertants of *fla18* that regained the ability to swim at 32?C. Following UV mutagenesis, 46 independent swimming strains were isolated. A dCAPS marker that distinguishes the polymorphism in wild-type and *fla18* was used to analyze 8 of the 46 revertants/suppressors. The restriction enzyme *Mbo*II cuts the wild-type PCR product, but not the *fla18* PCR product. A representative gel of the PCR and digest is shown in Figure?1. Digestion by *Mbo*II indicates that these eight strains are likely to be true revertants. The restoration of the original codon was confirmed in these eight revertants by Sanger sequencing (Table?4). Thus, our revertant analysis indicates that mutation in the *FLA10* kinesin is the causative mutation in *fla18*. To examine the temperature-sensitive phenotype of the *fla18* mutant strain, it was shifted from the permissive temperature of 21?C to the restrictive temperature of 32?C, and the percentage of flagellated cells was determined. While wild-type cells remain approximately 80% flagellated, the *fla18* cells lose their flagella gradually and at 7 hours most cells are aflagellate (Figure?2A). The length of the flagella was measured and the length changed by only 2 ?m (Figure?2B). The cells may be losing their flagella by detachment rather than by shortening.

**Figure 1:**
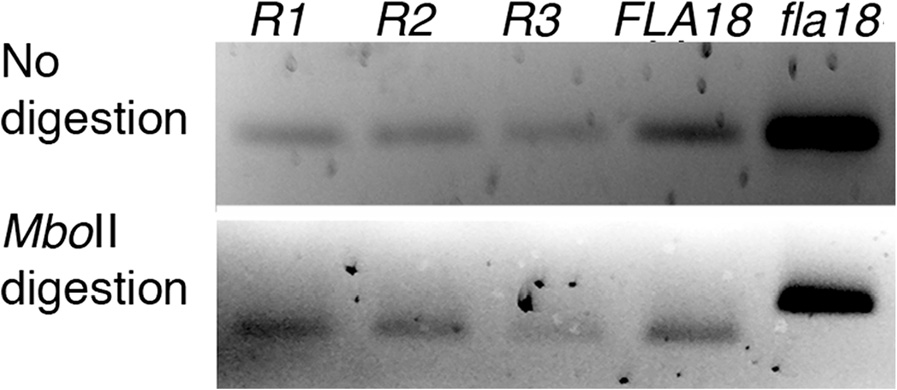
**PCR assays to provide evidence for identifying the *fla18 *gene by reversion.** A dCAPS marker shows reversion of *fla18*. Upper panel, a 132 bp PCR fragment was amplified in wild-type, *fla18*, and three *fla18* true revertants (*R1*, *R2*, and *R3*). Lower panel: the amplified fragment was subjected to *Mbo*II digestion, which generates a 103 bp fragment if the enzyme site is present in the PCR product.

**Table 4:** **Reversion of *fla18 *** **allele provides evidence of causality**

**Strain name**	***Mbo*** **II**	**Sequence for digestion (underline)**^**a**^	**Amino acid**	**Sanger sequence**
Wild-type	Cut	GA**A**GAAGGCAGAT	Glutamic acid	G**A**G
*fla18*	Not cut	GA**G**GAAGGCAGAT	Glycine	G**G**G
*R1*	Cut	GA**A**GAAGGCAGAT	G to E	G**G**G to G**A**G
*R2*	Cut	GA**A**GAAGGCAGAT	G to E	G**G**G to G**A**G
*R3*	Cut	GA**A**GAAGGCAGAT	G to E	G**G**G to G**A**G
*R5*	Cut	GA**A**GAAGGCAGAT	G to E	G**G**G to G**A**G
*R6*	Cut	GA**A**GAAGGCAGAT	G to E	G**G**G to G**A**G
*R7*	Cut	GA**A**GAAGGCAGAT	G to E	G**G**G to G**A**G
*R8*	Cut	GA**A**GAAGGCAGAT	G to E	G**G**G to G**A**G
*R9*	Cut	GA**A**GAAGGCAGAT	G to E	G**G**G to G**A**G

^a^Underlined nucleotides in the third column indicate the recognition site. This site is created by the primers in the wild-type (*FLA18*) sequence but not in the mutant (*fla18*) sequence.

**Figure 2:**
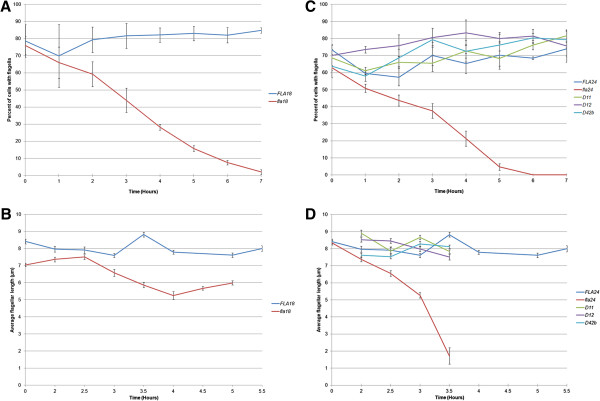
**Flagellar loss and shortening of *fla18 *and *fla24 *at the restrictive temperature. (A,C)** After transfer to the restrictive temperature, the percentage of flagellated cells was determined at 1-hour intervals by counting 200 cells in triplicate. **(B,D)** Flagellar length was measured at the indicated intervals after shifting cells to the restrictive temperature and measuring 100 flagella. Bars indicate the standard error of the mean.

### *FLA24* encodes the cytoplasmic dynein heavy chain

The *fla24* allele was mapped to chromosome 6 near the mating-type locus [31] and linkage to the mating-type locus was confirmed in an additional 243 tetrads (239:0:4; PD:NPD:T). There is only one change in *fla24* in the mapped interval after subtracting changes found in other unrelated strains [53]. The candidate in the interval is DHC1b, the cytoplasmic dynein gene for retrograde IFT (Table?2). The T to C mutation predicts a L_3242_P change. We used a PCR-based assay to examine linkage of the flagellar phenotype with the alteration in the cytoplasmic dynein gene. The PCR product generated is 303 bp long in both the *fla24* and wild-type strains. The mutant product is cut by *Nci*I but not cut by *Alw*N1, while the wild-type product is cut by *Alw*N1 but not by *Nci*I. This change cosegregates with the flagellar assembly defect in 59 meiotic progeny. To ask if this change is responsible for the phenotype, we again used reversion/suppressor analysis. Following mutagenesis, 64 independent swimming strains were recovered. Nine of the strains are no longer cut by *Nci*I. Of these, *Alw*N1 fails to cut three (Figure?3, Table?5). Sanger sequencing verified that we recovered six true revertants that changed the proline at amino acid 3243 back to leucine, and three pseudorevertants that changed the proline to either serine (2) or to arginine (1).

**Figure 3:**
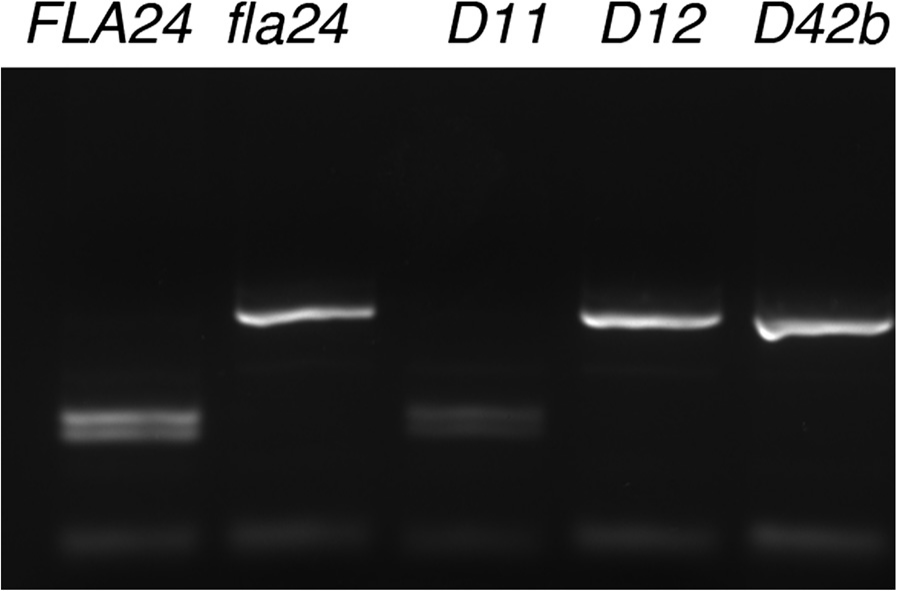
**PCR assays to provide evidence for identifying the *fla24 *gene by reversion.** A dCAPS marker shows reversion of *fla24*. A 303 bp PCR fragment was amplified in wild-type (*FLA24)*, *fla24*, and three *fla24* revertants (*D11*, *D12*, and *D42b*). The amplified fragment was subjected to *Alw*NI digestion, which generates three fragments of 51, 120, and 133 bps if the enzyme site is present in the PCR product as in wild-type and the true revertant (*D11*), or two fragments of 51 and 273 bps in the *fla24* mutant and pseudorevertants (*D12* and *D42b*).

**Table 5:** **Reversion of *fla24 *** **allele provides evidence of causality**

**Strain name**	***Alw*** **NI**	***Nci*** **I**	**Sequence for digestion (underline)**^**a**^	**Amino acid**	**Sequence**
*fla24*	Not cut	Cut	CAGCTGCCGGG	Proline	C**C**G
Wild-type	Cut	Not cut	CAGCTGCTGGG	Leucine	C**T**G
*D7*	Cut	Not Cut	CAGCTGCTGGG	P to L	C**C**G to C**T**G
*D9b*	Cut	Not Cut	CAGCTGCTGGG	P to L	C**C**G to C**T**G
*D11*	Cut	Not Cut	CAGCTGCTGGG	P to L	C**C**G to C**T**G
*D12*	Not cut	Not Cut	CAGCTGTCGGG	P to S	C**C**G to **T**CG
*D25b*	Cut	Not Cut	CAGCTGCTGGG	P to L	C**C**G to C**T**G
*D37*	Cut	Not Cut	CAGCTGCTGGG	P to L	C**C**G to C**T**G
*D42b*	Not cut	Not Cut	CAGCTGCGGGG	P to R	C**C**G to C**G**G
*D43*	Not cut	Not Cut	CAGCTGTCGGG	P to S	C**C**G to **T**CG
*D57*	Cut	Not Cut	CAGCTGCTGGG	P to L	C**C**G to C**T**G

^a^The underlined nucleotides in the fourth column indicate the recognition site for *Nci*I or *Alw*NI.

Currently, there are five other mutant alleles identified in the cytoplasmic dynein gene in *Chlamydomonas*. Three show nonconditional phenotypes; they assemble very short flagella [9,10], and two temperature-sensitive alleles have been identified. The *dhc1b*^*ts*^ allele assembles one-half length flagella (5.5 to 6 ?m) at the permissive temperature of 18?C. Upon shifting cells to the restrictive temperature, the flagella shorten by about one-half in 2.5 hours and are very short by 24 hours [32]. The *dhc1b-3* allele shows a very slow decrease in flagellar length and number upon shifting from 21?C to 34?C [27]. It takes nearly 4 days for the cells to shorten their flagella. We determined the number of cells with flagella in the *fla24* allele (Figure?2C). In contrast to the other conditional alleles, the *fla24* allele shows much faster shortening and loss of flagella. By 2.5 hours, there is noticeable shortening, and by 6 hours the population is aflagellate (Figure?2D). The true revertant and two pseudorevertants (P to S and P to R) remain flagellated at the restrictive temperature over the same time period as would be expected (Figure?2C).

### DHC1b and D1bLC are reduced in *fla24* at the restrictive temperature

DHC1b localizes to the basal body region and along the flagella in both wild-type and *fla24* at 21?C. At this temperature, the signal intensities at the basal body and in the flagella are comparable between wild-type and *fla24* cells (Figure?4A). In the *dhc1b-3* allele, the levels of DHC1b and the dynein light intermediate chain D1bLIC are reduced when cells were shifted to 34?C [27]. After the shift to 32?C, the wild-type cells maintain the same localization and similar signal intensity of DHC1b throughout the assay (5 hours). However, the intensity of DHC1b at the basal body and flagella in *fla24* cells starts to show a reduction within 2 hours, before the majority of cells lost their flagella (Figures?2 and 4). The DHC1b signal remains detectable in the basal body area, but decreases with time at the restrictive temperature. By immunoblots, we showed that DHC1b is reduced by 18-fold in *fla*24 flagella compared to wild-type flagella (Figure?4C). In contrast, the three revertants (D11, D12, and D42b) show similar signal intensities and locations to wild-type cells at both the permissive and restrictive temperatures (Figure?4B and Additional file 2: Figure S1).

**Figure 4:**
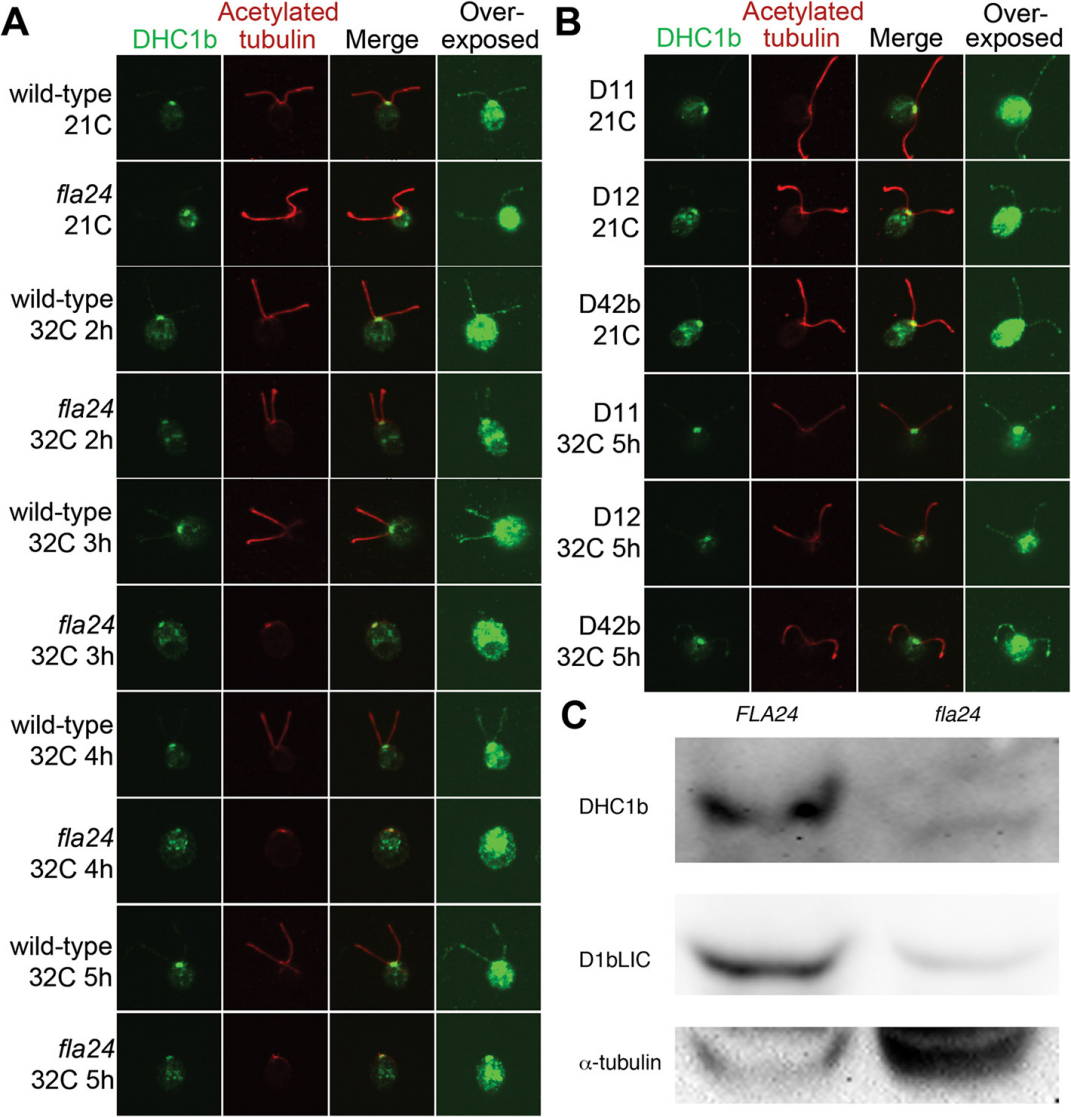
**Localization of DHC1b in wild-type, *fla24*, and *fla24 *revertants at 21?C and 32?C.** DHC1b staining is shown in green (first column) and the flagella are labeled with acetylated ?-tubulin (red, second column). Merged images of both staining are shown in the third column. Overexposed DHC1b signals are shown in the forth column to show the localization of DHC1b in the flagella. Cells were obtained from 21?C and various time points at 32?C, as indicated. **(A)** Wild-type and *fla24* cells. **(B)***fla24* revertants. **(C)** Twenty micrograms of flagellar proteins were isolated from wild-type (*FLA24*) and *fla24* cells at 21?C, and probed with DHC1b and D1bLIC antibodies. The membrane that used to probe D1bLIC was stripped and reprobed with ?-tubulin to normalize the loading.

The localization of D1bLIC in wild-type cells appears similar to that of DHC1b. The signal intensities of D1bLIC remain high in wild-type cells at 21?C and for at least 5 hours after cells are shifted to 32?C (Figure?5A). However, in *fla24* cells, even though D1bLIC shows similar localization, the signal intensity is greatly reduced when compared to wild-type cells at 21?C (Figure?5A). The signal shows further reduction as cells lose their flagella (Figure?5A, 32?C, 2 to 5 hours). The level of D1bLIC is reduced, as shown by immunoblots of flagellar proteins at 21?C by 16-fold (Figure?4C). An immunoblot of D1bLIC using whole cell extract from wild-type and *fla24* cells at 21?C indicates that similar to flagellar D1bLIC, the amount of D1bLIC in whole cell extract is reduced approximately 16-fold in the mutant (Figure?5C). We also observed approximately 3-fold reduction when wild-type cells were switched from 21?C to 32?C for 5 hours (Figure?5C). Consistent with the observation by immunofluorescence (Figure?5A), we were unable to detect the D1bLIC signal by immunoblot after *fla24* cells were shifted to 32?C for 5 hours (Figure?5C). Similar to observation of DHC1b, the signal intensities of D1bLIC remain high in all three revertants (Figure?5B and Additional file 3: Figure S2).

**Figure 5:**
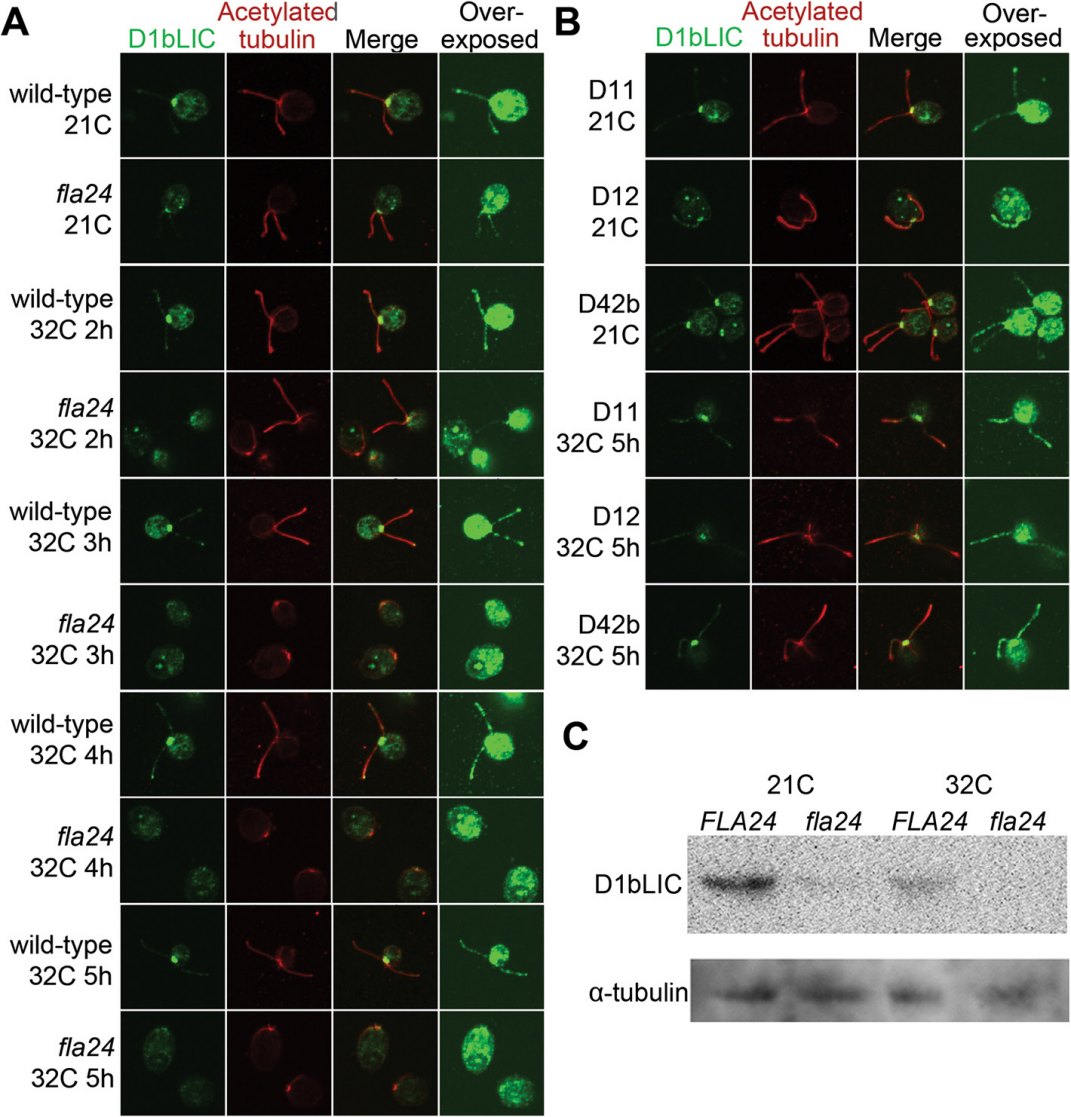
**Localization of D1bLIC in wild-type, *fla24*, and *fla24 *revertants at 21?C and 32?C.** D1bLIC staining is shown in green (first column) and the flagella are labeled with acetylated ?-tubulin (red, second column). Merged images of both staining are shown in the third column. Overexposed D1bLIC signals are shown in the forth column to show the localization of D1bLIC in the flagella. Cells were obtained from 21?C and various time points at 32?C, as indicated. **(A)** Wild-type and *fla24* cells. **(B)***fla24* revertants. **(C)** Twenty micrograms of whole cell protein extract were isolated from wild-type (*FLA24*) and *fla24* cells at both 21?C and 32?C, and probed with the D1bLIC antibody. The membrane was then stripped and reprobed with ?-tubulin to normalize the loading.

### Localization of IFT81 is perturbed at permissive temperature in *fla24* but not in *fla18*

At the permissive temperature of 21?C, Iomini *et al*. found that *fla18* IFT trains show a reduced anterograde velocity, and the *fla24* IFT particles show a reduced retrograde velocity [11]. These mutant strains have defects in the anterograde and retrograde motors, respectively. Therefore, we asked the whether the localization of IFT81, a complex B protein, is affected in these strains.

In the *fla18* mutant strain at the permissive temperature, no change of localization or reduction of intensity of IFT81 was observed when compared to wild-type cells (Figure?6). After cells were shifted to the restrictive temperature, the IFT81 signals remained in the basal body region and in the flagella until the cells became aflagellate at 6 hours. IFT81 appears unperturbed by the *fla18* mutation.

**Figure 6:**
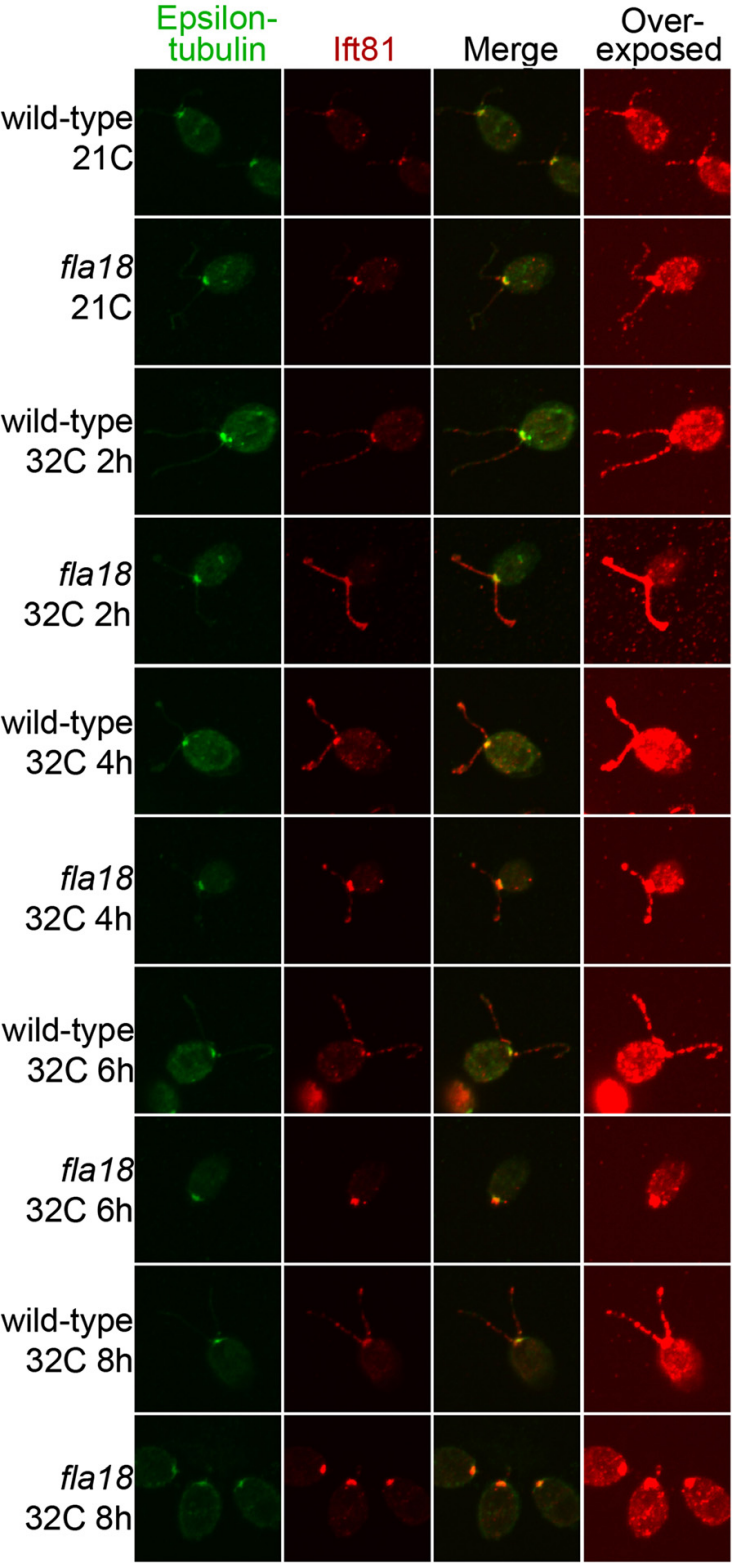
**Localization of IFT81 in wild-type and *fla18 *cells at 21?C and 32?C.** Staining of ?-tubulin (green), IFT81 (red), merged images, and overexposed IFT81 signals are shown. Cells were obtained from 21?C and various time points at 32?C, as indicated.

In the *fla24* mutant strain at the permissive temperature, the majority of the IFT81 protein is not localized at the basal body as observed in wild-type and the other mutants, but accumulates in the proximal ends (0.4 to 1.4 ?m; n = 20) of flagella regardless of their flagellar length (Figure?7A). This accumulation remains unchanged after cells were shifted to restrictive temperature (32?C) until flagella are lost. In the true revertant (D11) of *fla24*, IFT81 localizes to the basal body region and flagella at both temperatures, as observed in wild-type cells (Figure?7B and Additional file 4: Figure S3). While the localizations of IFT81 in the two pseudorevertants (D12; P_3243_S and D42b; P_3243_R) at permissive temperature are identical to the pattern in wild-type cells, they show accumulation of IFT81 at the proximal ends of flagella after 6 hours and 4 hours at the restrictive temperature (Figure?7B and Additional file 4: Figure S3), respectively. This suggests that the serine or arginine at the conserved leucine position does not completely restore function.

**Figure 7:**
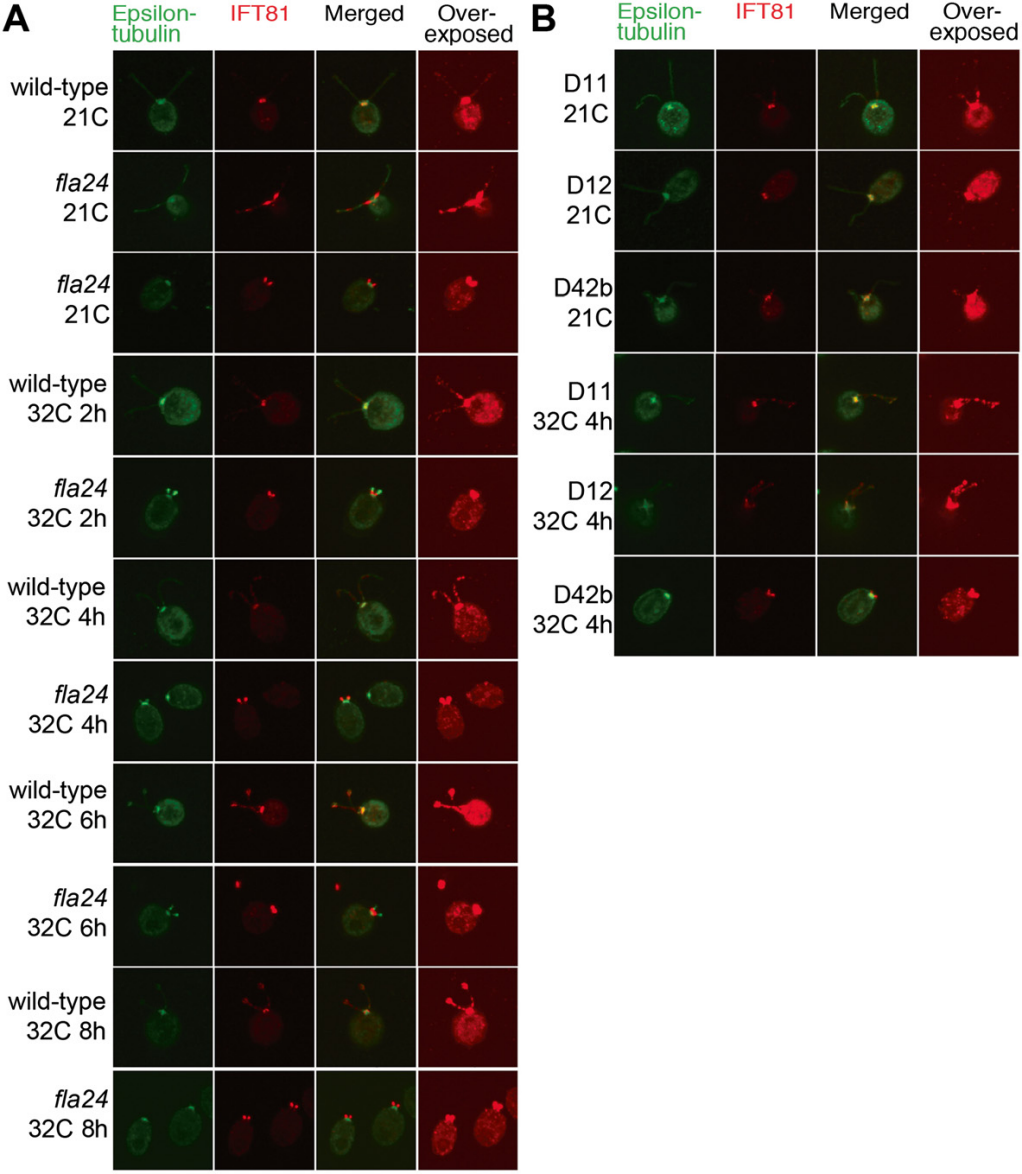
**Localization of IFT81 in wild-type, *fla24, *and *fla24 *revertants at 21?C and 32?C.** Staining of ?-tubulin (green), IFT81 (red), merged images, and overexposed IFT81 signals are shown. Cells were obtained from 21?C and various time points at 32?C, as indicated. **(A)** Wild-type and *fla24* cells. **(B)***fla24* revertants.

## Discussion

Conditional mutants have been extremely useful in the study of essential genes in many cellular processes from ribosome assembly to cell division to secretion to synaptic vesicles. A collection of temperature-sensitive flagellar assembly mutants in *Chlamydomonas* has allowed the analysis of intraflagellar transport; they have documented that anterograde movement requires kinesin and IFT complex B, and that retrograde movement requires cytoplasmic dynein and IFT complex A. In addition, remodeling of IFT trains at the tip requires IFT172, a protein in complex B, and a temperature-sensitive mutation in this protein leads to a retrograde defect (Table?1). Of the 21 extant conditional mutants, previous work together with this work has identified the causative lesion in 12 of them. All of these genes encode either IFT components or motor proteins (Table?1). Six have mutations in one of the three kinesin motor genes and four other mutations are in the cytoplasmic dynein motor. This bias suggests that conditionality may be more easily achieved in the motor subunits than in the IFT components.

Whole genome sequencing in *Chlamydomonas* has been fruitful when the gene is mapped to a region or chromosome [30]. We have developed a collection of changes in other wild-type and mutant strains that can be used to eliminate non-causative candidates [53]. In addition, the transcriptional profiles during regeneration of flagella are useful for identifying candidate flagellar assembly genes [54]. For *fla18*, we narrowed the list of candidates from 892 to 85 (Table?2) by combining the data from whole genome sequencing and transcriptional profiles. If we had used this strategy for *fla24,* only three of the 62 genome-wide candidates showed increased levels of transcript during regeneration. For *fla9* (unpublished data at *Cilia*)*,* this strategy would have narrowed the 78 genome-wide candidates to eight. The use of both data sets may help to obviate the need for genome-wide fine mapping of flagellar assembly mutants.

The mutation in *fla18* affects the same amino acid that is mutated in the *fla10-14* strain*.* In *fla10-14*, the glutamic acid is changed to lysine but in *fla18* (now renamed *fla10-16*) it becomes a glycine. The two alleles have different kinetics of flagellar loss [8]; the E_24_K allele takes over 12 hours to see loss of 50% of the flagella compared to the E_24_G allele that takes only 6 hours to see complete loss (Figure?2A). This glutamic acid is conserved in all kinesin-2 molecules across the ciliated phylogenetic tree (n = 75, data not shown). As speculated previously [8], it seems likely that this amino acid may interact with the P-loop and be important for motor activity. Interestingly, the *fla2* mutant shows a 'fragile? phenotype [24]. Upon shifting cells to the restrictive temperature, the flagella detach rather than shorten. We observe similar detachment with the *fla18* allele. Since *fla18* greatly reduced anterograde IFT velocity, it is interesting to speculate that either a component is transported that maintains the integrity of the flagellar axoneme or a signal to maintain the integrity fails. This phenotype is allele-specific, which supports the idea that different alleles may provide different information about the functions of anterograde IFT.

The *fla1* mutation (now *fla8-2*) and the *fla8-1* mutation are in the second motor subunit of kinesin-2 [8]; they were thought to affect different phases of IFT [11]. The *fla10-1* and *fla8-1* alleles show similar phenotypes with normal anterograde velocity but a reduced ratio of anterograde to retrograde particles, while *fla8-2* and *fla18* show similar phenotypes with reduced anterograde velocity and a reduced ratio of anterograde to retrograde particles. Different mutations have different phenotypic effects on IFT.

*fla24* is a mutation in the cytoplasmic dynein. The mutant *fla24* allele has several helpful properties that will allow dissection of a successful IFT cycle. The IFT trains must be assembled at the basal bodies, turn around at the tip to change from anterograde to retrograde movement, and then be reloaded at the base for anterograde transport. In our study of *fla15* (IFT144) and *fla17* (IFT139) IFT complex A mutants, we observed that diploid cells heterozygous for *fla24* and either *fla15* or *fla17* were aflagellate at 32?C but flagellated at 21?C [31]. It is not unexpected that defects in complex A might show an enhancement of a cytoplasmic dynein mutant phenotype.

Since *fla24* is compromised by reduced mutant Complex B proteins, we considered that the IFT dynein function may also be sensitized to dynein inhibitors since the retrograde velocity is reduced to 0.9 ?m/second from 3.1 ?m/second for wild-type cells and the frequency of retrograde particles is reduced [11]. Ciliobrevin D is a small molecule that inhibits cytoplasmic dynein [55]. We asked if *fla24* cells were more sensitive to ciliobrevin D than wild-type cells. Surprisingly, with the addition of 100 ?M ciliobrevin D, *fla24* cells showed no effect on flagellar length after 30 minutes (data not shown), although this concentration has been shown to reduce the retrograde particle frequency after 5 minutes [56]. Further experiments to examine IFT particle rates with the mutant and inhibitor will shed more light on synthetic interactions.

Upon the shift of *fla24* cells to 32?C, the flagella are lost within 4 hours (Figure?2C); this is quite rapid compared to the other DHC1B alleles. The amount of DHC1b in flagella is greatly reduced as observed by immunofluorescence and immunoblot (Figure?4). The *fla24* cells have three interesting phenotypes at 21?C that may suggest roles for the AAA5 domain of cytoplasmic dynein. First, the retrograde velocity and number of particles are reduced [11]. Second, the level of light intermediate chain (D1bLIC) is reduced as indicated by immunofluorescence and immunoblots (Figures?4C and 5). Third, the IFT81 protein distribution is dramatically altered; instead of localizing to the basal body and along the flagella, IFT81 has left the basal body region and is concentrated in the proximal approximate 1 ?m of the flagella (Figure?7A). Unlike the *dhc1b-3* allele that shows a reversal in the direction of phototaxis [27], we never observed a change in the phototaxis phenotype of the *fla24* mutant over a 6-hour period (data not shown). Again, it is clear that different alleles have different phenotypic properties.

The cytoplasmic dynein molecule is composed of a central ATP-hydrolyzing ring that has six AAA modules arranged around the ring?s central pore. The *fla24* mutation falls into an alpha-helix in the AAA5 domain that is extended into the alpha helical strut/buttress [57,58] (Figure?8). The strut/buttress is postulated to have a high degree of plasticity that may be important for its function in communicating between the microtubule-binding domain (MTBD) of the stalk and the AAA ring. The distal region of the strut interacts with the middle of the stalk and a deletion of the distal end of the strut removes this interaction. Allosteric communication between AAA1 and MTBD is postulated to be relayed through the C-sequence, the strut, and the stalk [57].

**Figure 8:**
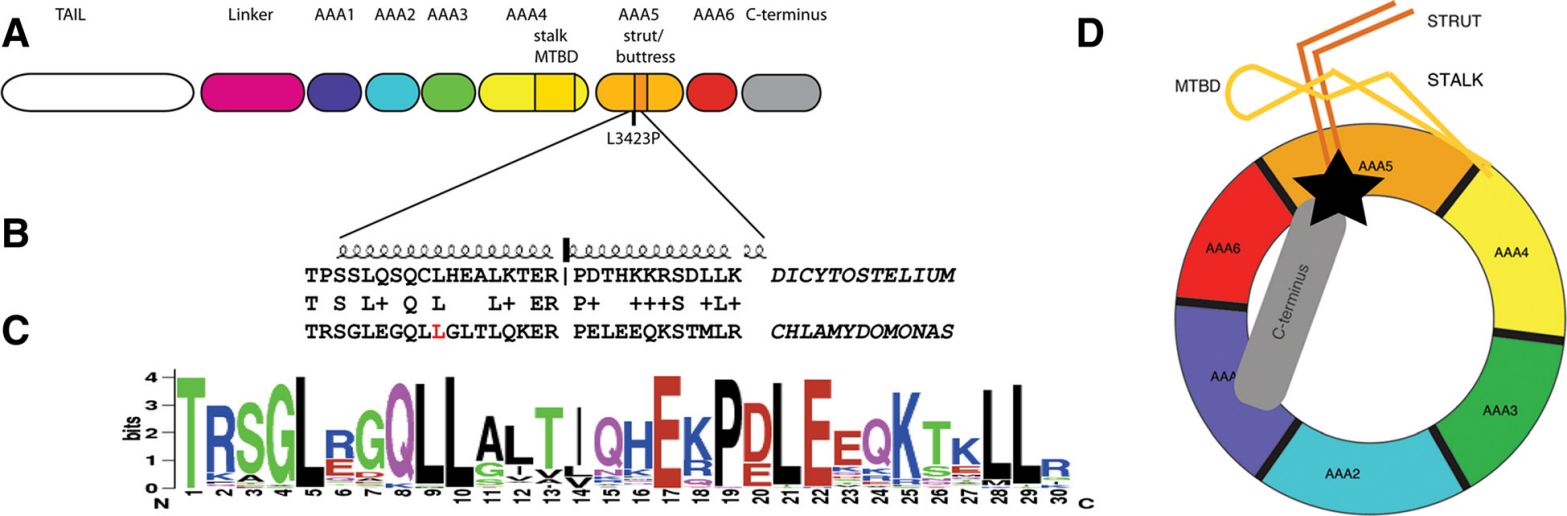
**The *fla24 *mutation falls in a conserved alpha-helix that leads to the strut/buttress structure. (A)** Diagram of domain in the cytoplasmic dynein based on the structure of *Dictyostelium* cytoplasmic dynein [50]. The stalk is extended from AAA4 and contains the microtubule-binding domain (MTBD) (in darker yellow). The strut or buttress is extended from AAA5. **(B)** Alignment of *Dictyostelium* cytoplasmic dynein and *Chlamydomonas* cytoplasmic dynein 1b in the AAA5 domain into the strut. A leucine (L_3243_) is mutated in the *fla24* allele. The alpha helices above the alignment show the extent of the helices in the *Dictyostelium* structure. **(C)** Logo of the region in panel B from 52 organisms [59] showing conservation based on height. L_3243_ at position 10 in the logo is conserved in all 52 organisms. **(D)** Diagram (redrawn from Pazour *et al*. [50]) showing the back side of the ring that may allow information to flow between the AAA1 domain and the MTBD through the C-terminus, strut, and stalk. L_3243_ (indicated by a black triangle) may be critical for this communication. MTBD, microtubule-binding domain.

Two in-frame deletions of 6 and 7 amino acids in the cytoplasmic dynein of *Neurospora crassa* affect the strut [60]. The 3739 ?6 amino acid deletion removes part of the first coil of the strut and is postulated to affect communication between the MTBD and the nucleotide status of the AAA1 domain. The dynein localizes distally in this mutant. The 3756 ?7 amino acid deletion is in the first coil of the strut as well but causes aggregation of the dynein. It is postulated that this mutation may lock the structure of the dynein. The cytoplasmic dynein mutants in *Neurospora* also demonstrate that different alleles have very different phenotypic effects [52].

The reduced retrograde velocity of IFT trains [31], the reduction in D1bLIC at the basal bodies, and the accumulation of IFT81 in the *fla24* mutant suggest that the mutant has a defect in moving along the microtubules and in remodeling the IFT trains at the proximal end of the flagella via cargo binding. In our screen for suppressor and revertants of *fla24*, we have identified at least three genes that suppress the restrictive temperature flagellar assembly defect. These strains may provide information about interactions with the dynein heavy chain that will speak to how the strut and other structures communicate flagellar assembly defect (data not shown).

## Conclusions

Whole genome sequencing provides a fast and inexpensive means to identify chemically induced mutations in *Chlamydomonas*[30,53]. Identification of the remaining seven temperature-sensitive mutations will be greatly helped by this technique. The identification of multiple mutant alleles in kinesin and in cytoplasmic dynein that have different phenotypes will greatly help studies of the function of these proteins. The *fla18* allele is unique among the motor mutants in that it appears to deflagellate after the temperature shift instead of shortening. The *fla24* allele results in an abnormal localization of IFT81 near the basal bodies at the permissive temperature, which may suggest a defect in recycling IFT trains.

## Abbreviations

dCAPS: Degenerate cleaved amplified polymorphic sequence; DIC: Differential interference contrast; DTT: Dithiothreitol; HEPES: 4-(2-hydroxyethyl)-1-piperazineethanesulfonic acid; HRP: Horseradish peroxidase; IFT: Intraflagellar transport; iTRAQ: Isobaric tags for relative and absolute quantitation; KAP: Kinesin-associated protein; mu: Map unit; MTBD: Microtubule-binding domain; NIH: National institutes of health; PBST: Phosphate-buffered saline tween; PCR: Polymerase chain reaction; PVDF: Polyvinylidene fluoride; RNAi: RNA interference; SNP: Single nucleotide polymorphism; UV: Ultraviolet.

## Competing interests

The authors declare that they have no competing interests.

## Authors? contributions

HL performed the analysis of the whole sequence, analysis of dynein subunit localization, and immunoblots. NPN measured flagellar length and number. AJA prepared and examined the samples for immunofluorescence, and examined the effect of ciliobrevin D. SH mapped meiotic progeny from crosses of *fla24* x *FLA24*. SKD conceived of the study, performed the reversion analysis and PCR, and wrote the manuscript. All authors read and approved the final manuscript.

## Supplementary Material

Additional file 1: Table S1 Primers for reversion analysis of *fla18* and *fla24.* Click here for file

Additional file 2: Figure S1 Localization of DHC1b in *fla24* revertants at 32?C. Staining of DHC1b (green), acetylated ?-tubulin (red), merged images, and overexposed DHC1b signals are shown. Cells were obtained from various time points at 32?C, as indicated. Click here for file

Additional file 3: Figure S2 Localization of D1bLIC in *fla24* revertants at 32?C. Staining of D1bLIC (green), acetylated ?-tubulin (red), merged images, and overexposed D1bLIC signals are shown. Cells were obtained from various time points at 32?C, as indicated. Click here for file

Additional file 4: Figure S3 Localization of IFT81 in *fla24* revertants at 32?C. Staining of ?-tubulin (green), IFT81 (red), merged images, and overexposed IFT81 signals are shown. Cells were obtained from various time points at 32?C, as indicated. Click here for file
