# Supplementary figures and images for: New mutations in flagellar motors identified by whole genome sequencing in Chlamydomonas
Source: Cilia. 2013 Oct 30;2:14. doi: 10.1186/2046-2530-2-14 (PMC4132587; doi:10.1186/2046-2530-2-14)

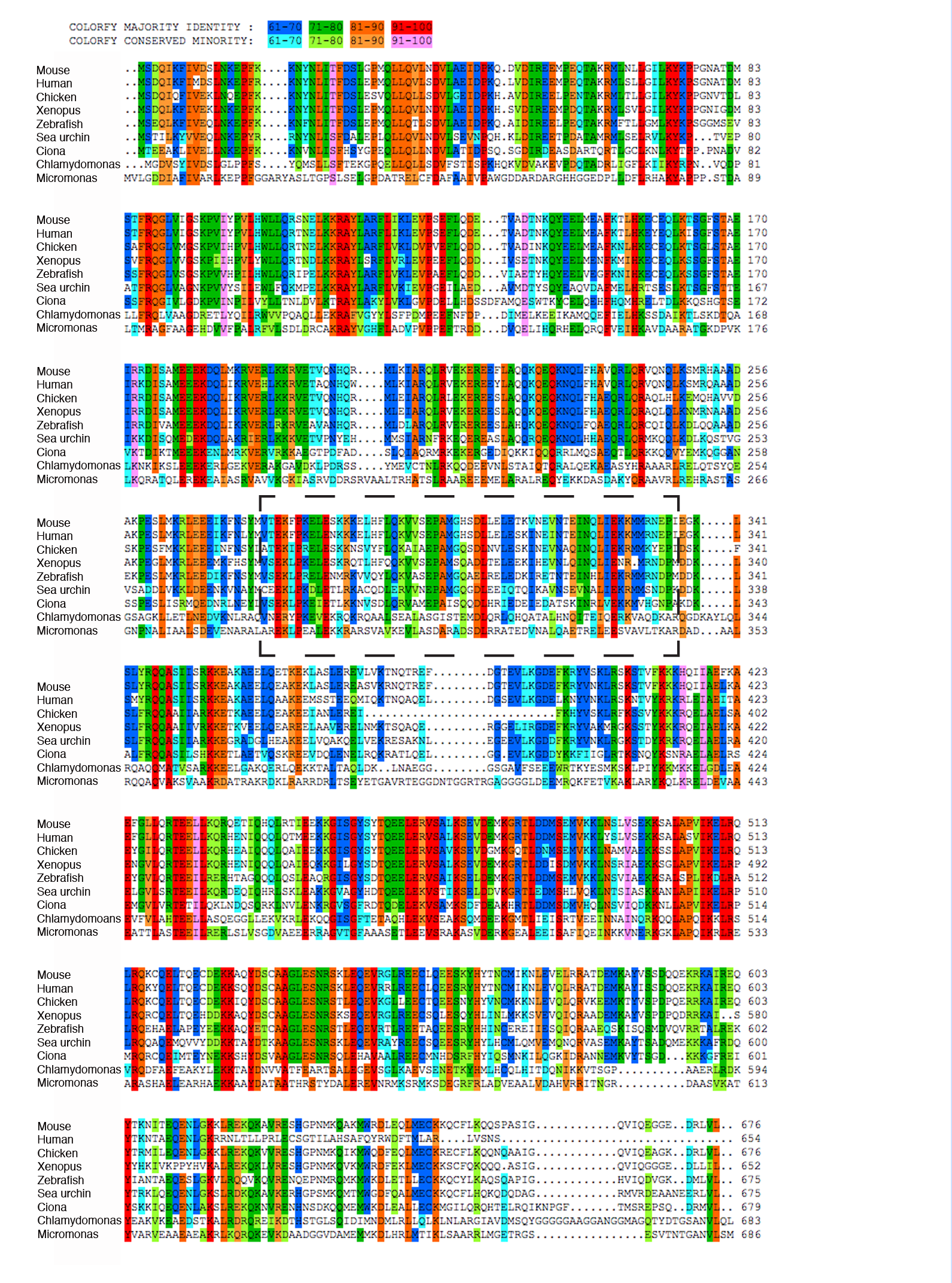

Supplement: Additional file 1: Table S1 — Primers for reversion analysis of fla18 and fla24. [file 2046-2530-2-14-S1.tif]

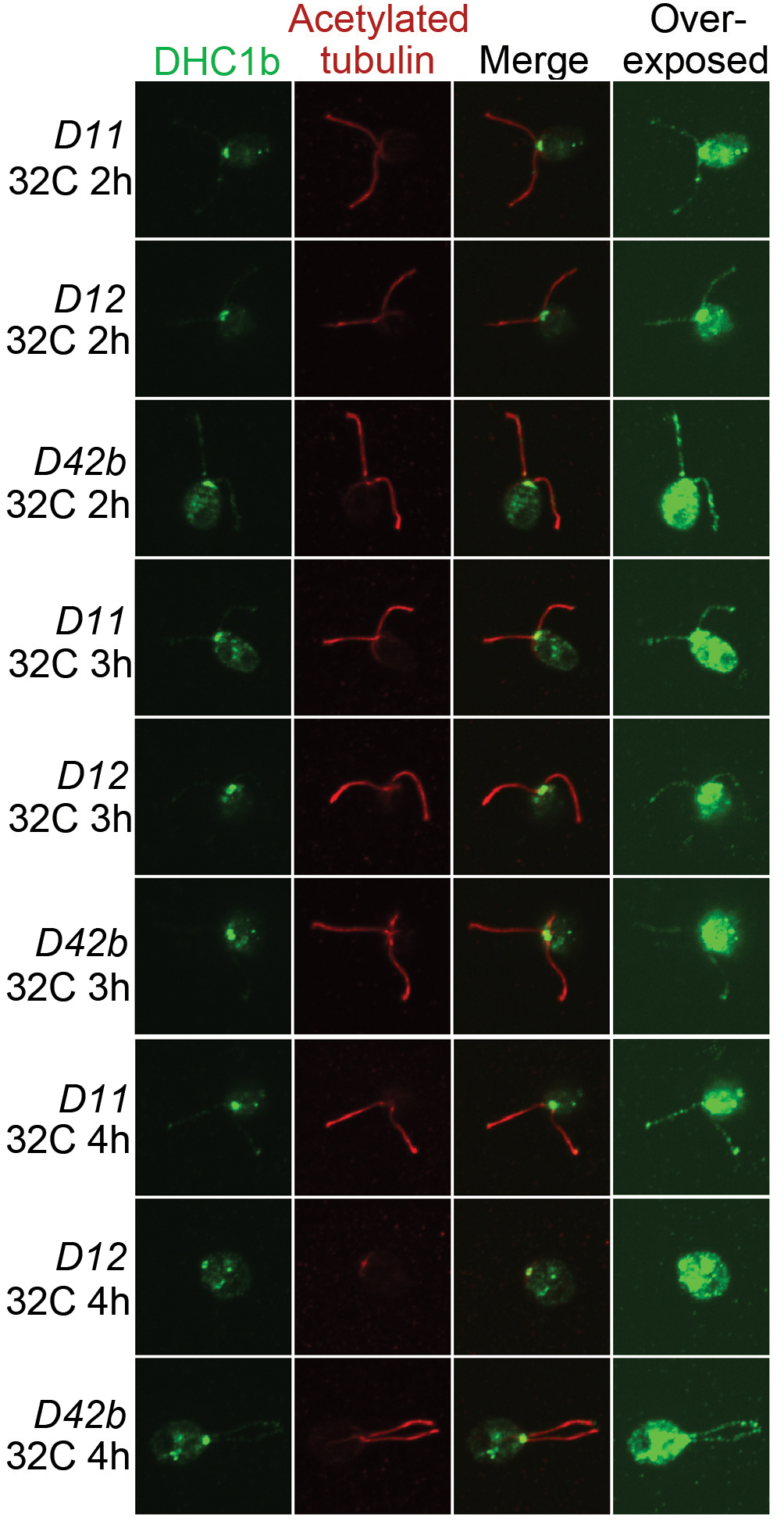

Supplement: Additional file 2: Figure S1 — Localization of DHC1b in fla24 revertants at 32?C. Staining of DHC1b (green), acetylated ?-tubulin (red), merged images, and overexposed DHC1b signals are shown. Cells were obtained from various time points at 32?C, as indicated. [file 2046-2530-2-14-S2.jpg]

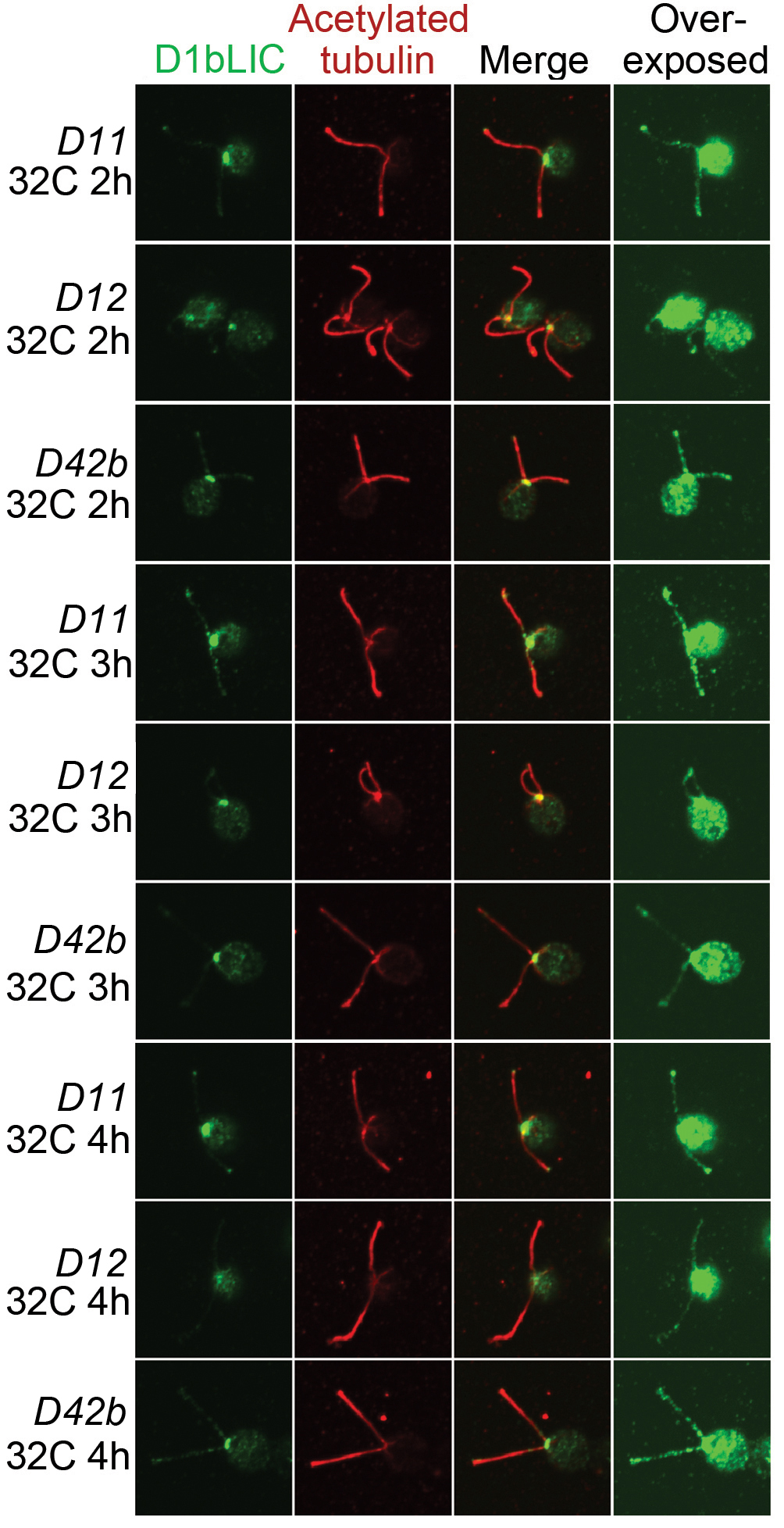

Supplement: Additional file 3: Figure S2 — Localization of D1bLIC in fla24 revertants at 32?C. Staining of D1bLIC (green), acetylated ?-tubulin (red), merged images, and overexposed D1bLIC signals are shown. Cells were obtained from various time points at 32?C, as indicated. [file 2046-2530-2-14-S3.jpg]

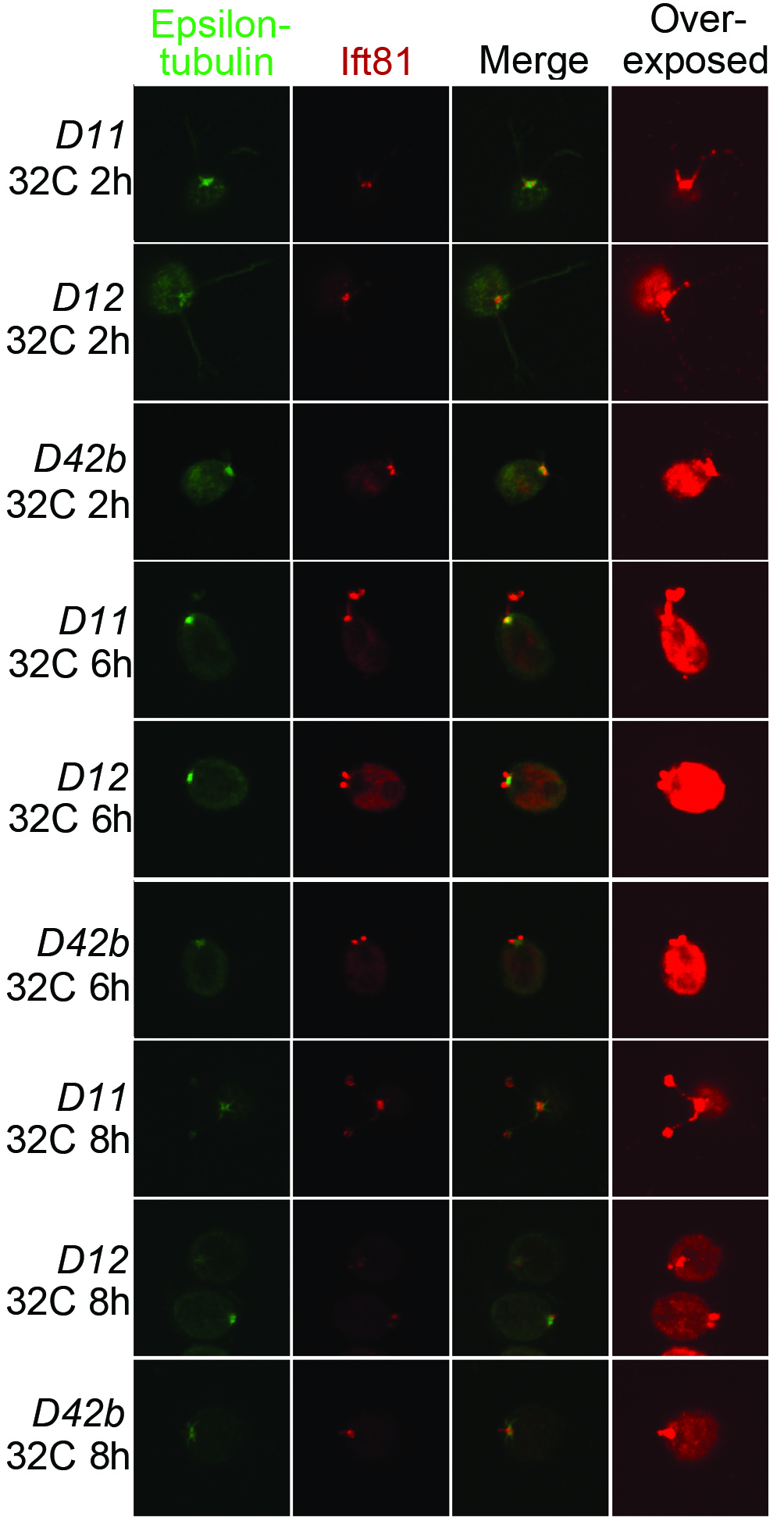

Supplement: Additional file 4: Figure S3 — Localization of IFT81 in fla24 revertants at 32?C. Staining of ?-tubulin (green), IFT81 (red), merged images, and overexposed IFT81 signals are shown. Cells were obtained from various time points at 32?C, as indicated. [file 2046-2530-2-14-S4.jpg]
